# A Temporal Threshold for Formaldehyde Crosslinking and Fixation

**DOI:** 10.1371/journal.pone.0004636

**Published:** 2009-02-27

**Authors:** Lars Schmiedeberg, Pete Skene, Aimée Deaton, Adrian Bird

**Affiliations:** Wellcome Trust Centre for Cell Biology, University of Edinburgh, Edinburgh, United Kingdom; University of Birmingham, United Kingdom

## Abstract

**Background:**

Formaldehyde crosslinking is in widespread use as a biological fixative for microscopy and molecular biology. An assumption behind its use is that most biologically meaningful interactions are preserved by crosslinking, but the minimum length of time required for an interaction to become fixed has not been determined.

**Methodology:**

Using a unique series of mutations in the DNA binding protein MeCP2, we show that in vivo interactions lasting less than 5 seconds are invisible in the microscope after formaldehyde fixation, though they are obvious in live cells. The stark contrast between live cell and fixed cell images illustrates hitherto unsuspected limitations to the fixation process. We show that chromatin immunoprecipitation, a technique in widespread use that depends on formaldehyde crosslinking, also fails to capture these transient interactions.

**Conclusions/Significance:**

Our findings for the first time establish a minimum temporal limitation to crosslink chemistry that has implications for many fields of research.

## Introduction

Chemical crosslinking with formaldehyde and related reagents is widely used to fix sub-cellular structures for microscopy and to immobilise protein-DNA contacts for chromatin immunoprecipitation (ChIP [1]–[4]). Exposure of living cells to formaldehyde results in covalent linkage with exposed amino and imino groups (notably in lysine and arginine sidechains). This forms a Schiff's base that can participate in a second linkage, creating methylene bridges between amino acids that were in close proximity (∼2 Å) in the native protein. Crosslinks between proteins and DNA and RNA are also possible, for example via the amino group on cytosine, though the difficulty in detecting these suggests that intra- and inter-protein crosslinks are far more abundant [2].

An assumption behind the widespread use of crosslinking is that the fixed structures accurately reflect molecular relationships in the living cell. In the present study, we have found that this assumption becomes invalid when intermolecular contacts are short-lived. This limitation to formaldehyde crosslinking became apparent via our studies of the methylated DNA binding protein MeCP2, which associates in a DNA methylation-dependent manner with heterochromatic foci in mouse cell nuclei [5], [6]. A series of mutants of the MeCP2 DNA binding domain fail to localize to heterochromatin in fixed cells, but localize indistinguishably from wildtype protein when living cells are examined by fluorescence microscopy. An equivalent discrepancy between living and formaldehyde-treated cells was seen at the level of ChIP, as the immunoprecipitated mutant proteins recovered little DNA compared with wildtype protein. Using Fluorescence Recovery After Photobleaching (FRAP), we showed that all mutants residing in heterochromatin for less than 2.5 seconds on average escape capture by crosslink chemistry and misleadingly indicate lack of localization. Our findings indicate that there is a minimum time required for formaldehyde fixation of protein DNA interactions, below which interpretation of ChIP and microscopy becomes problematical. This limitation to crosslinking as an experimental tool is likely to be general.

## Results

**Figure 1:**
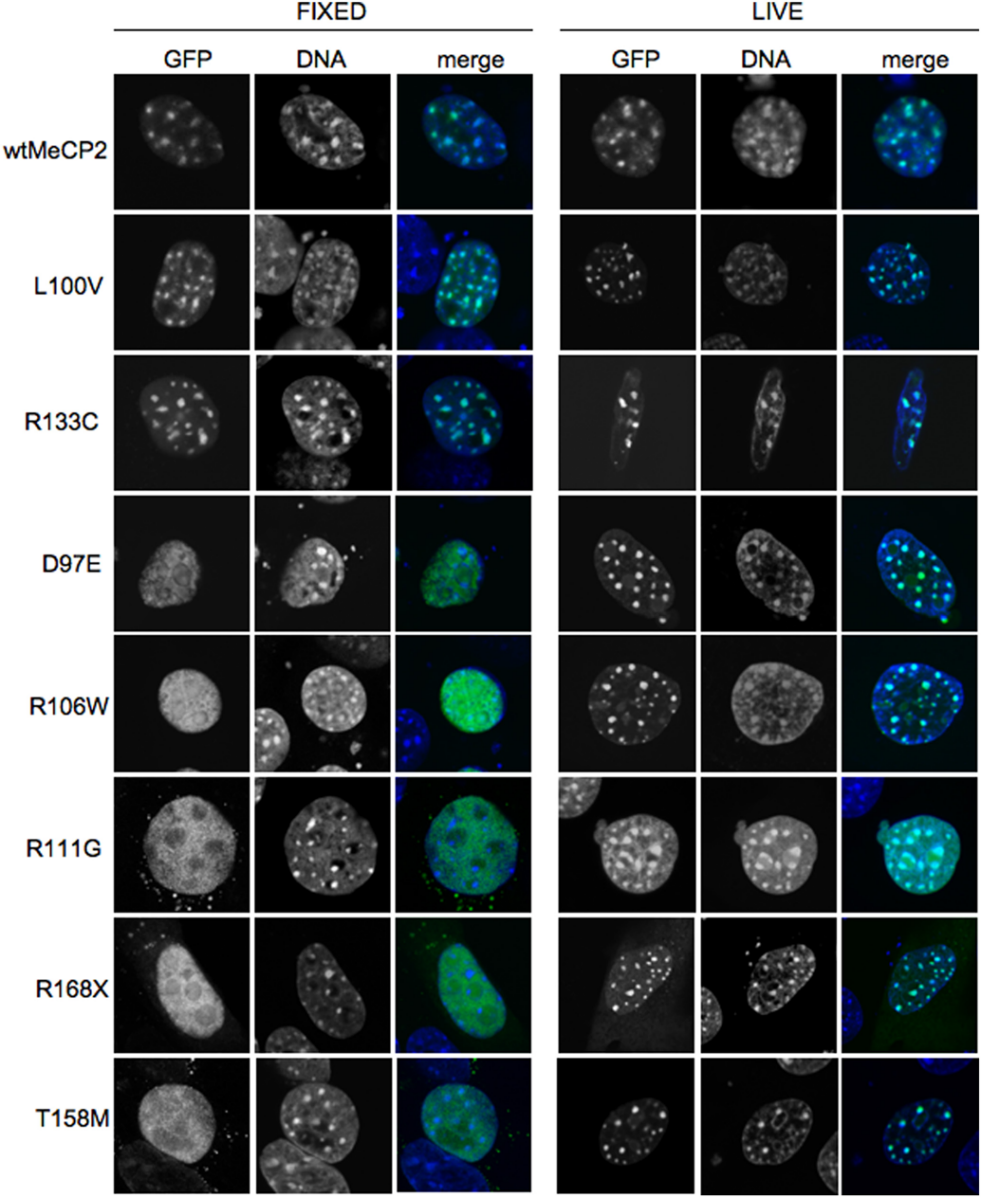
Major differences in nuclear localisation of MeCP2-GFP in living versus paraformaldehyde-fixed mouse fibroblasts. All MeCP2 mutants (labelled left) localized to nuclear foci corresponding to peri-centromeric heterochromatin in living cells, but many showed diffuse nuclear staining in the same cells after paraformaldehyde-fixation.

During a study of the dynamics of MeCP2 binding to chromatin, we transfected *Mecp2*-null fibroblasts with constructs expressing a range of mutant MeCP2 proteins fused to GFP. The mutations affected the DNA binding domain and were initially identified as causes of the neurological disorder Rett Syndrome [7]. As reported previously [8], many of these mutations prevent the localization of MeCP2 to densely methylated heterochromatic foci and give diffuse staining in fixed cells (Figs. 1, left). Two mutants (T158M and D97E) gave rise to a mixed population of punctate and diffusely stained nuclei. Surprisingly, when live cells were observed, all of the mutants, including those that showed diffuse nuclear staining in fixed cells, gave punctate localization indistinguishable from wildtype (Fig. 1, right). We conclude that the preferential heterochromatic localization seen *in vivo* has not been fixed by formaldehyde crosslinking.

**Figure 2:**
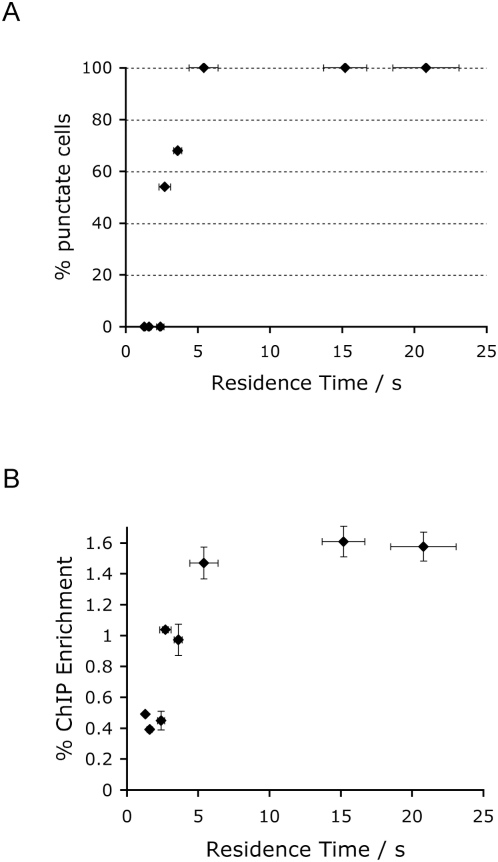
Inverse relationship between MeCP2 residence time on heterochromatin and the ability to crosslink this interaction by formaldehyde. A) In vivo residence times on heterochromatin of MeCP2 mutants were determined by FRAP. Mutants with a residence time above 5 seconds were 100% localized in fixed cells, whereas those with shorter residence times localized partially or not at all. B) Immunoprecipitation of formaldehyde-crosslinked MeCP2 is inefficient when the residence time is below 4 seconds. The mutants in order from left to right in panels A and B are: R168X, R106W, R111G, D97E, T158M, L100V, R133C and wildtype. Error bars on both axes correspond to ±standard deviation.

Fluorescence Recovery After Photobleaching (FRAP) was used to deduce the residence half-time (t_50_[S]) for each mutant protein on heterochromatic foci. A single focus was bleached and the time of taken for recovery of fluorescence due to replacement by protein from outside the bleached area was measured. Wildtype MeCP2 exchanged on average every ∼15 seconds [9], whereas most mutants showed reduced residence times [10]. When t_50_[S] values were plotted against the percentage of nuclei showing punctate staining, it emerged that all mutants showing diffuse staining in fixed cells had a residence time of less than 2.5 seconds, whereas all fully localized proteins had residence times in excess of 5.4 seconds (Fig 2A). Interestingly, the two partially localized mutants had intermediate t_50_[S] values between 2.5 and 4 seconds. The results demonstrate an inverse relationship between time spent bound to heterochromatin and the efficiency with which this liaison could be chemically fixed.

Is the apparent delocalization of MeCP2 mutant forms that have short chromatin residence times also seen at the level of ChIP? To test this, NIH3T3 cells were transfected with the mutant series, crosslinked with formaldehyde and sheared chromatin was immunoprecipitated with an anti-GFP antibody. The efficiency of recovery of major satellite DNA, which is the major component of centromeric heterochromatin, corresponded closely with the microscopy results (Fig. 2B). Mutants with a residence time of 5.4 seconds or more were efficiently immunoprecipitated, whereas rapidly exchanging mutants gave significantly weaker signals. The two mutants with residence times of 2.5–4 seconds showed intermediate ChIP recovery. Once again there is a discrepancy between the strong and consistent localisation of mutant MeCP2 to heterochromatin as seen by microscopy *in vivo* and the relatively poor association that is detectable by ChIP.

## Discussion

The results show that proteins which predominantly localize to chromatin in living cells can seem weakly chromatin-associated by both microscopy and ChIP if the rate of exchange between bound and unbound protein is rapid. In the case of MeCP2, the threshold above which formaldehyde fixation is effective corresponds to a binding time of ∼5 seconds. Comparable mutational series that affect the duration of intermolecular binding have not been identified for other proteins. As a result, it is not possible to determine the generality of the 5 second threshold identified for MeCP2. It is highly likely, however, that the ability to cross-link other DNA binding proteins is also limited by the intrinsic temporal constraints on crosslink chemistry. For example, the dynamic association between glucocorticoid receptor and cognate binding sites is refractory to formaldehyde crosslinking [11]. Photobleaching studies of the association between NF-kappaB and an array of high affinity sites *in vivo* established exchange with a t_50_[S] of ∼1 s, which may also escape fixation with formaldehyde [12]. These examples emphasise that many protein-DNA interactions that are transient but biologically important would probably escape detection by ChIP.

A potential explanation for our findings is that the fixation reaction that covalently links MeCP2 to heterochromatic DNA requires an average of 5 seconds of protein immobility to complete. Wildtype MeCP2 remains bound for ∼15 seconds [9] and is therefore almost quantitatively cross-linked to chromatin. As a result, the image of wildtype MeCP2 localisation by either microscopy or ChIP accurately reflects reality. On the other hand, mutants that dissociate from chromatin in less than 5 seconds fail to undergo covalent crosslinking to chromatin and appear to be predominantly nucleoplasmic by both assays. Solomon and Varshavsky [2] found that purified DNA binding proteins were indetectably crosslinked to DNA *in vitro*, suggesting that protein-DNA crosslinks are far less frequent than inter- and intra-molecular protein crosslinks. Formaldehyde-ChIP may therefore depend upon topological trapping of DNA via crosslinking of histones and associated proteins, rather than covalent linkages to DNA itself. We propose that intramolecular protein crosslinking involving sidechain amino groups and amide nitrogens of the peptide bond can explain the apparent nucleoplasmic accumulation of mutant MeCP2 molecules. Intramolecular crosslinking will proceed in the presence of formaldehyde whether the protein is chromatin bound or in a free unbound state. Eventually, we suggest, the accumulation of internal covalent linkages prevents the unbound protein from associating again with DNA by locking it in a rigid inert configuration.

The literature contains anomalies that can potentially be explained by the inability of formaldehyde to fix transient interactions. For example, the localisation of the nucleosome binding proteins known as High Mobility Group proteins B and N (HMGBs and HMGNs) during the cell cycle is controversial. Live cell imaging shows that exogenous GFP-tagged HMGNs are predominantly associated with chromatin during mitosis, but images of fixed cells indicate that the proteins are dispersed throughout the nucleoplasm at this stage [13], [14]. It was shown that HMGBs exchange dynamically with chromatin, but the residence time was not determined. Based on the present findings, we suggest that HMGBs associate with chromatin too transiently for formaldehyde fixation to crosslink HMGN/B proteins in their native chromatin-bound state.

A further discrepancy that might be attributable to failure of formaldehyde fixation concerns the *in vivo* distribution of chromatin binding sites for Polycomb Group (PcG) proteins. Results using formaldehyde-ChIP technology were compared with a study that used “DamID” to locate the sites. DamID involves expression at approximately physiological levels of a fusion between PcG and the Dam DNA methyltransferase. Recruitment of PcG causes the DNA methyltransferase to covalently mark *in vivo* DNA sites where PcG is bound. Over 60% of PcG-bound sites detected by DamID were not detected by the ChIP studies. This may be because the interaction of PcG with DNA is too transient to be efficiently crosslinked by formaldehyde. Measurements of PcG exchange rates on *Drosophila* polytene chromosome bands indicated t_50_[S] values of 1–10 minutes, which is one to two orders of magnitude longer than the minimum duration of binding needed to fix MeCP2 [15]. It was notable, however, that the majority of fluorescent PcG signal recovered in a few seconds after bleaching. This was considered to reflect diffusion of unbound PcG into the bleached area, but may correspond to a “fast” exchanging fraction in addition to the “slow” fraction that was emphasised. A fraction of this kind may exchange with chromatin too rapidly for capture by formaldehyde fixation.

## Materials and Methods

### Construction of plasmids

A his-tagged expression plasmid of wildtype MeCP2 fused to EGFP wtMeCP2 was constructed by cloning a PCR amplified EcoRI/BamHI fragment into the pEGFP-c1 vector as described [16]. MeCP2 mutants were subsequently generated by site directed mutagenesis using mismatch primers according to manufacturer's protocol (Stratagene). Primer sequences are available on request. All constructs were verified by sequencing.

### Cell culture and transfection

*Mecp2* ^−/y^ fibroblasts [17] were cultured in DMEM supplemented with 10% FCS, glutamine and penicillin/streptomycin in 5% CO_2_ at 37°C. For photobleaching experiments, cells were seeded onto 25 mm coverslips. Between 16 and 20 h before starting the experiment, cells were transfected with GFP fusion constructs using polyethylenimine according to manufacturers protocol (JetPei, Qbiogene).

### Photobleaching studies

For photobleaching studies, transfected cells were grown on coverslips and 16–20 h after transfection and then mounted on a Leica SP2 TCS AOBS confocal scanning microscope equipped with a heated stage and an environmental CO_2_-chamber (Incubator S, Pecon). FRAP was performed with the 453 nm, 488 nm, 496 nm and 513 nm lines of an argon-neon laser with a nominal output of 8 mW using a 63× HCX PL Apo NA 1.4 oil objective. Images with an acquisition time of 0.344 s were collected before (10 images) and after (1000 images) bleaching a spot of 4 µm^2^ for one second. Each independent transfection experiment was performed in triplicate and 10–15 cells were photobleached in each preparation. For imaging, the laser intensity was attenuated to 4% of nominal output. The t_50_[S] was calculated by using the formula t_50_[S] = t((F∞−F_0_)*0.5)−t(F_0_), where F_0_ is the fluorescence minimum at t_0_ (first image after the bleach) and F∞ is the fluorescence maximum at the end of the measurement [18].

### MeCP2 Localisation

For analysis of fixed material, transfected cells were grown on 8-well slides and 16–20 h after transfection were washed 2× with PBS and incubated with 4% paraformaldehyde for 10 minutes at room temperature. After a further two washes with PBS, preparations were mounted in Vectashield containing DAPI (Vector Laboratories). MeCP2 localisation was determined by counting 50–100 cells on each slide. For localization studies in living cells, transfected cultures were maintained at 37°C and incubated with 9 µM Hoechst33342 (Sigma) for 15 min before acquiring images with a 405 nm diode laser and a 488 nm argon laser using a 63× HCX PL Apo Na 1.4 oil objective on a Leica SP2 confocal microscope. All experiments were performed in triplicate.

### ChIP for exogenously expressed GFP-MeCP2 fusions

NIH 3T3 cells were cultured and transfected as above. Typically 2×10^6^ cells were trypsinized and pelleted at 330 g for 5 min at room temperature. The cells were washed in phosphate-buffered saline (PBS) and then crosslinked in 1% formaldehyde in PBS for 10 min at room temperature. The crosslinking was stopped with the addition of glycine to 125 mM for 5 min at room temperature. The cells were pelleted, washed in PBS, lysed in 1% SDS, 10 mM EDTA, 50 mM Tris-HCl pH 8.0 for 10 min on ice and then diluted 1∶10 in dilution buffer (1% Triton X-100, 2 mM EDTA, 150 mM NaCl, 20 mM Tris-HCl pH 8.0). The chromatin was sonicated for 3 min at 30% amplitude, using a digital sonifier (Branson). Precipitated debris was removed by centrifugation at 16,000 g for 10 min at 4°C. Fragmented chromatin was pre-cleared for 1 h at 4°C with tRNA/BSA/protein A sepharose beads. Chromatin immunoprecipitations were performed using 2 µg Invitrogen anti-GFP A11122 antibody overnight at 4 C. We chose to use the anti-GFP antibody rather than anti-MeCP2 to avoid precipitation of endogenous MeCP2 in the transfected mouse cells. To isolate the immunocomplexes, 50 µl of protein A sepharose were added to the samples for 1 h at 4°C. The beads were then washed once in buffer 1 (0.1% SDS, 1% Triton X-100, 2 mM EDTA, 150 mM NaCl, 20 mM Tris-HCl), four times in buffer 2 (0.1% SDS, 1% Triton X-100, 2 mM EDTA, 500 mM NaCl, 20 mM Tris-HCl), once in buffer 3 (250 mM LiCl, 1% NP-40, 1% deoxycholate, 1 mM EDTA, 10 mM Tris-HCl) and three times in TE buffer (10 mM Tris-HCl, 1 mM EDTA). Immunocomplexes were eluted with 200 µl extraction buffer (1% SDS, 100 mM NaHCO_3_) and crosslinks were reversed by adding 5 M NaCl to a final concentration of 300 mM and incubation at 65°C overnight. DNA was phenol extracted, ethanol precipitated and resuspended in 200 µl 0.1×TE. Real-time PCR was carried out with iQ SYBR Green Supermix (Bio-Rad) on an iCycler (Bio-Rad) according to the manufacturer's instructions using the following steps: 95°C 3 min denaturation, followed by 95°C for 30 s, 62°C for 30 s, 72°C for 30 s for a total of 45 cycles. Major satellite was amplified using primers: GGCGAGAAAACTGAAAATCACG; AGGTCCTTCAGTGTGCATTTC. Chromatin samples were also analysed by western blotting to verify comparable expression of the transgenes (not shown). All experiments were performed in triplicate using independent biological material.
